# Uncommon Occurrence of Pulmonary Aspergillosis Caused by Aspergillus sydowii: A Case Report

**DOI:** 10.7759/cureus.51353

**Published:** 2023-12-30

**Authors:** Akimichi Nagashima, Tadashi Nagato, Tomoko Kobori, Minoru Nagi, Yasumi Okochi

**Affiliations:** 1 Department of Respiratory Medicine, Japan Community Health Care Organization Tokyo Yamate Medical Center, Tokyo, JPN; 2 Department of Fungal Infection/Antimicrobial Resistance Research Center, National Institute of Infectious Diseases, Tokyo, JPN

**Keywords:** fungi, bronchiectasis, aspergillus spp, aspergillus sydowii, pulmonary aspergillosis

## Abstract

This case report presents an unusual occurrence of pulmonary aspergillosis caused by *Aspergillus sydowii* in a 26-year-old male patient. The patient is from Nepal and had no significant medical history and was previously in good health. Chest computed tomography (CT) scans revealed localized bronchiectasis primarily in the left inferior lingular segment and the left lower lobe. Subsequently, bronchial lavage fluid was collected, and a comprehensive culture examination was conducted to confirm the cause of the infection. While *Aspergillus* *fumigatus* typically predominates as the cause of pulmonary aspergillosis, our bronchial lavage fluid culture revealed the presence of a filamentous fungus, identified as *Aspergillus sydowii* through molecular analysis. Thus, we conclusively identified this particular strain of fungus as the etiological factor behind the patient’s condition. Notably, pulmonary aspergillosis due to *Aspergillus sydowii* is exceedingly rare, and we present this case alongside relevant prior data for comprehensive clinical insight. This case underscores the clinical significance of *Aspergillus sydowii* as a fungal pathogen, emphasizing the importance of early recognition and managing fungal infections.

## Introduction

This case report describes a rare condition: pneumonia and bronchiectasis due to *Aspergillus sydowii* in an adult patient. Pulmonary aspergillosis refers to the clinical spectrum of lung disease caused by several *Aspergillus* spp [1,2]. Although there are hundreds of species of *Aspergillus* in nature, only a few of them can cause infection in patients. Among these, *A. fumigatus* is the most common cause of infections, followed by *A. flavus* and *A. niger* [2,3]. Despite advances in diagnostic methods and therapy, mortality rates remain high, especially in severely immunosuppressed subjects [1]. *Aspergillus sydowii* is a filamentous fungus that is commonly found in marine environments. While it is known to cause a range of infections in marine animals [4], its role as a human pathogen has not been extensively studied.

We performed a literature search for the previously published case of *A. sydowii*. However, to our best knowledge, there is no report of *A. sydowii* infection in lungs with bronchiectasis. Our findings will provide valuable insights into the management of these respiratory infections.

## Case presentation

We describe the case of a 26-year-old man from Nepal who was previously in good health. An abnormal shadow was detected by chest X-ray at the previous clinic. The patient reported no fever, but presented with slight cough without wheezing, sputum production or dyspnea. Physical examination of the skin, abdomen, and cardiac exam were normal ranges. The patient had a history of bacterial pneumonia at age 12, but no other medical history. He had no recent travel, no exposure to environmental toxins, and no history of immunosuppressant or steroid use prior to the onset of symptoms.

**Figure 1:**
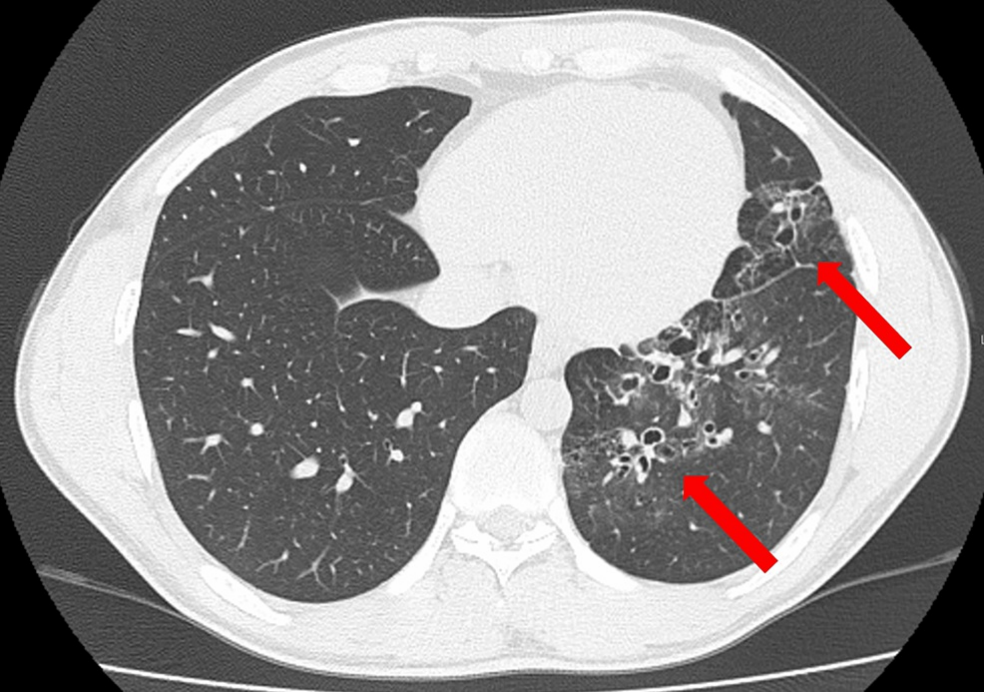
Chest CT Lung window CT revealed infiltrates, gland grass opacities and bronchodilation in the left inferior lingular segment and the left lower lobe (arrows).

His initial blood test showed white blood cells (WBCs) of 5,700/µL and C-reactive protein (CRP) of 0.2 mg/dL. Anti-*Aspergillus* antibody, *Aspergillus* antigen and β-D glucan were not elevated in the blood test (Table 1). A chest CT scan revealed faint infiltrates, ground glass opacities, bronchial wall thickening and bronchiectasis in the left inferior lingular segment and the left lower lobe (Figure 1).

**Table 1:** Laboratory values

Parameter	Value	Reference range
White blood cell count	5.7 × 10^3^/µL	3.5 – 9.0 × 10^3^/µL
Neutrophils	36. 3%	37.0 – 72.0%
Eosinophils	4.9%	0.0 – 5.0%
Hemoglobin	15.7 g/dL	14.0 – 18.0 g/dL
Platelet count	26.9 × 10^4^/µL	12.0 – 36.0 × 10^4^/µL
Creatinine	0.76 mg/dL	0.65 – 1.07 mg/dL
Blood urea nitrogen	8 mg/dL	8 – 20 mg/dL
Sodium	141 mEq/L	135 – 145 mEq/L
Potassium	4.7 mEq/L	3.4 – 5.0 mEq/L
Albumin	4.6 g/dL	3.9 – 4.9 g/dL
C-reactive protein (CRP)	0.2 mg/dL	0.0 – 0.4 mg/dL
Total Immunoglobulin E (IgE)	332 IU/mL	≦250 IU/mL
Beta-D glucan	≦4 pg/mL	<11.0 pg/mL
Aspergillus galactomannan	Negative	Negative
Anti-Aspergillus antibody	Negative	Negative
T-SPOT.TB test	Negative	Negative

**Figure 2:**
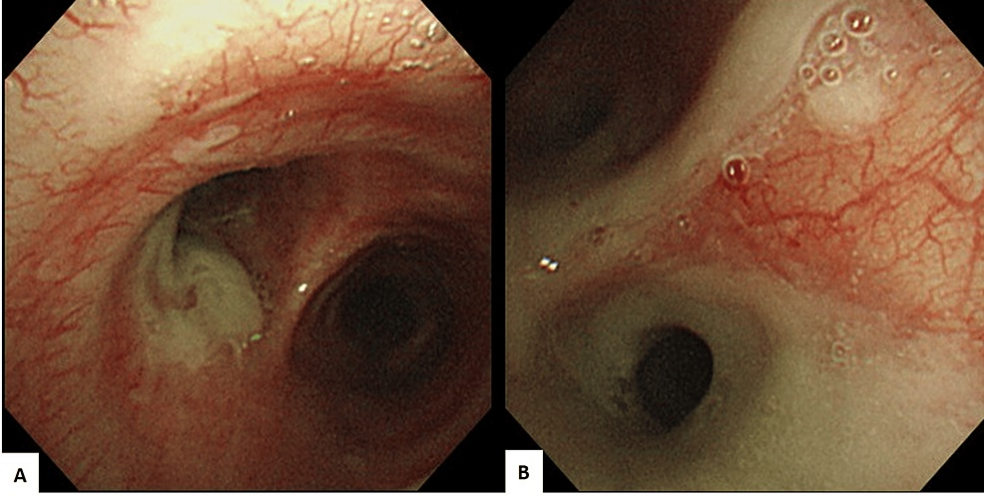
Left-bronchus bronchoscopy The image obtained yellowish plaques in the left main bronchus (A) and the left lower lobe bronchus (B).

**Figure 3:**
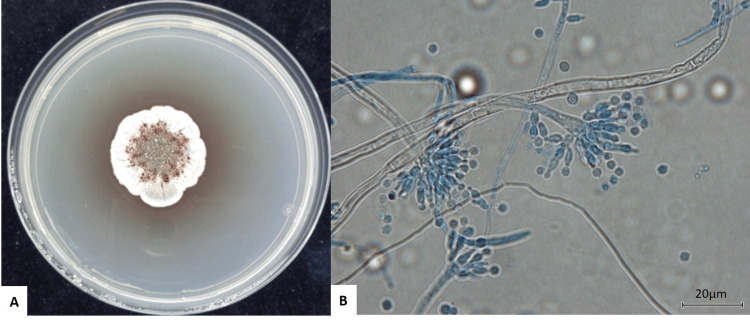
Morphology of Aspergillus sydowii. Overview of the growth of *Aspergillus sydowii*. The cultures were incubated on potato dextrose agar at 30℃ for 14 days. Pictures were taken on day 14. (A) Macroscopic morphology. (B) Microscopic morphology (lactophenol cotton blue staining, ×400).

Bronchoscopy was performed because the patient was unable to expectorate enough sputum for bacterial examination. Yellowish plaques were found in the left main bronchus and the left lower lobe bronchus, which were collected, and bronchial lavage fluid was obtained by bronchoscope (Figure 2A, 2B). Cultures of the sample yielded filamentous fungus, which exhibited atypical characteristics not consistent with commonly encountered *Aspergillus* species such as *A. fumigatus* or *A. flavus*. Colonies with brown centers and white margins were observed (Figure 3A). Microscopic pictures show round-shaped conidia and phialides covering the vesicle with long conidiophores (Figure 3B). Subsequently, a sample of the fungal colony was dispatched to the National Institute of Infection Diseases (NIID) in Japan for further comprehensive analysis. The molecular analysis targeting the internal transcribed spacer region and D1/D2 region of the rRNA gene and the β-tubulin gene identified the isolated fungus as *Aspergillus sydowii*. Notably, no other bacteria or alternative *Aspergillus* species were detected within the specimen.

Based on these results, we diagnosed that the patient was suffering from pulmonary aspergillosis due to *A. sydowii*. As we confirmed the minimum inhibitory concentration of voriconazole was 1.0 µg/mL, even though this value was based on the *in vitro* drug susceptibility test, the treatment with voriconazole was initiated with admission to observe the concentration of voriconazole. After some days of admission, the patient was discharged and continued therapy. Following voriconazole administration, the patient's progression was followed through continuous monitoring of the voriconazole plasma concentrations. Consequently, the ground glass opacities observed in chest CT were not improved after eight months, but the cough symptom was alleviated.

## Discussion

*Aspergillus* fungi are ubiquitous in the environment worldwide. *Aspergillus fumigatus*, in particular, has been associated with respiratory infections in humans. Invasive aspergillosis is a significant concern for patients with compromised immune systems and has exhibited an upward trend in incidence in recent years [5]. Fungal infections, including those caused by *Aspergillus*, are also a major problem among patients with chronic pulmonary diseases such as chronic obstructive pulmonary disease (COPD) and bronchiectasis, where the normal lung construction is compromised.

*Aspergillus sydowii* has been isolated from environmental samples from gorgonian communities in the Caribbean, the Ecuadorian Pacific, and coastal waters of Australia in marine ecosystems [4,6,7]. While it is known to cause infections in marine animals, pathogenicity in humans has not been fully studied. The diagnosis of *A. sydowii* infections can be challenging due to the non-specific symptoms and lack of reliable diagnostic tools. A previous study showed the frequency of *Aspergillus* spp. in respiratory samples of patients with *Aspergillus* infection [8]. Although *A. sydowii* was rarely isolated from respiratory samples in the study, the authors did not regard it as an etiologic pathogen. Moreover, according to a survey conducted in Canada, *A. sydowii *was found to be responsible for 0.49% of nail infections [9]. In our case, bronchial lavage fluid was collected and a sample was cultured, which yielded a colony that looks different from the common fungus causing pulmonary infections in humans. It is necessary to perform molecular analysis for accurate identification, although it may not be routinely performed because of its cost and facility limitation. However, *A. sydowii* is a cryptic species that cannot be identified without molecular identification [3,10]; therefore, it is recommended to perform molecular analysis, especially for *Aspergillus* species with uncommon colony characteristics.

The treatment of *Aspergillus* infections involves the use of azole antifungal agents, such as itraconazole, voriconazole, posaconazole, and isavuconazole [11]. Among these, itraconazole or voriconazole is typically introduced as initial therapy for pulmonary aspergillosis. Voriconazole is often favored due to its enhanced activity against *Aspergillus* and data suggesting better clinical outcomes and tolerability compared to itraconazole [12,13]. However, a meta-analysis of observational data revealed no significant difference in outcomes between itraconazole and voriconazole [14]. A prospective comparative study of these two agents might be necessary for a conclusive assessment. Voriconazole is associated with several unique adverse reactions, including transient vision changes, a photosensitivity rash, and periostitis, which is observed specifically in long-term users of voriconazole [11]. These symptoms should be closely monitored during follow-up. Additionally, follow-up imaging is one means of assessing progress of pulmonary aspergillosis. It is recommended to conduct follow-up imaging every three to six months after initiating antifungal treatment and then less frequently, or in the case of any major change in clinical status [15]. However, the optimal treatment and management for *A. sydowii* infections is not well-established, as there is limited data on the susceptibility of this fungus to antifungal agents and management methods. In this case, the patient was treated with voriconazole based on the results of the drug susceptibility test, and the symptoms of cough were relieved after several weeks of treatment. No significant adverse reactions were observed during follow-up.

In the pathophysiology of bronchiectasis, *Aspergillus* infections may directly damage the airways [16]. The spores of *Aspergillus* enter the lungs inhaled with air and begin to germinate [5]. The mycelium that develops in the lungs releases toxic metabolites, inhibiting the immune system and allowing further development of mycelium and inflammation in the respiratory tract. Studies have also shown that certain fungal metabolites, such as gliotoxin and fumagillin, can exacerbate damage to lung epithelial cells [17,18]. The continuation of inflammation disrupts the normal structure and leads to bronchiectasis [19]. Although we did not find clear evidence that *A. sydowii* produces gliotoxin and fumagillin as *A. fumigatus* does, especially in the respiratory tract in humans, some metabolites from *A. sydowii* may be associated with respiratory inflammation and bronchiectasis. Another possible mechanism involves conditions that induce bronchiectasis, such as cystic bronchiectasis, where *Aspergillus* causes secondary infection and inflammation [16]. In our case, the mechanism causing bronchiectasis remains obscured, but it is possible that *A. sydowii* was involved in its etiology or exacerbation. Overall, the mechanisms underlying bronchiectasis in *Aspergillus* infection are complex and not fully understood. Further studies are needed to elucidate the precise mechanisms involved and to develop more effective treatments for *Aspergillus*-induced bronchiectasis.

One potential limitation of this report is the possibility of an allergic reaction to *Aspergillus* that was not fully explored. While eosinophil levels in the blood were not elevated at the initial examination, other tests such as *Aspergillus*-specific immunoglobulin E (IgE), fractional exhaled nitric oxide (FeNO), pulmonary function tests, and skin prick test were not conducted. These tests may have provided additional insights into the nature and severity of the patient’s immune response to the fungal infection. It is therefore important to note that an allergic reaction to *Aspergillus* was not to be completely ruled out as a contributing factor to the patient’s respiratory symptoms.

## Conclusions

Our case report emphasizes the emerging clinical significance of *A. sydowii* as a fungal pathogen. The diagnosis of *A. sydowii* infections requires highly specific techniques to identify the species. Since we only detected the genetic data of *A. sydowii* from bronchial samples, we diagnosed respiratory infection due to *A. sydowii* in this case. This case underscores the importance of early recognition and management of fungal infections, even in patients without underlying lung diseases, and emphasizes the need for further research into the pathogenesis and optimal management of *Aspergillus* infections.
